# The Genomes of Four *Meyerozyma caribbica* Isolates and Novel Insights into the *Meyerozyma guilliermondii* Species Complex

**DOI:** 10.1534/g3.117.300316

**Published:** 2018-01-08

**Authors:** Leone De Marco, Sara Epis, Aida Capone, Elena Martin, Jovana Bozic, Elena Crotti, Irene Ricci, Davide Sassera

**Affiliations:** * School of Bioscience and Veterinary Medicine, University of Camerino, 62032, Italy; † Department of Biology and Biotechnology, University of Pavia, 27100, Italy; ‡ Department of Veterinary Science and Public Health, University of Milan, 20133, Italy; § Department of Food, Environmental and Nutritional Sciences, University of Milan, 20133, Italy

**Keywords:** *Meyerozyma caribbica*, Genome Report, *Drosophila suzukii*, *Culex quinquefasciatus*, *Anopheles stephensi*, *Aedes aegypti*

## Abstract

Yeasts of the *Meyerozyma guilliermondii* species complex are widespread in nature and can be isolated from a variety of sources, from the environment to arthropods to hospital patients. To date, the species complex comprises the thoroughly studied and versatile *M. guilliermondii*, the hard to distinguish *M. caribbica*, and *Candida carpophila*. Here we report the whole genome sequencing and *de novo* assembly of four *M. caribbica* isolates, identified with the most recent molecular techniques, derived from four Diptera species. The four novel assemblies present reduced fragmentation and comparable metrics (genome size, gene content) to the available genomes belonging to the species complex. We performed a phylogenomic analysis comprising all known members of the species complex, to investigate evolutionary relationships within this clade. Our results show a compact phylogenetic structure for the complex and indicate the presence of a sizable core set of genes. Furthermore, *M. caribbica*, despite a broad literature on the difficulties of discerning it from *M. guilliermondii*, seems to be more closely related to *C. carpophila*. Finally, we believe that there is evidence for considering these four genomes to be the first published for the species *M. caribbica*. Raw reads and assembled contigs have been made public to further the study of these organisms.

*Meyerozyma caribbica* (anamorph *Candida fermentati*) and *Meyerozyma guilliermondii* (anamorph *Candida guilliermondii*) are two closely related yeast species belonging to the *M. guilliermondii* species complex (Bai *et al.* 2000; Vaughan-Martini *et al.* 2005). *M. guilliermondii* has been object of several studies, with a broad bibliography describing its multiple interesting properties and applications (Papon *et al.* 2013), and is extensively used in biotechnology in a variety of tasks. *M. guilliermondii* is employed in riboflavin production (Tanner *et al.* 1945) and the bioconversion of xylose into xylitol (Zou *et al.* 2010), and is a promising source of enzymes (Gong *et al.* 2007) and biofuel (Wang *et al.* 2012).

Moreover, it is considered a killer yeast, having a broad range of antimicrobial activity against bacteria (Zhao *et al.* 2010), fungi (Coda *et al.* 2013), and even protozoa (Dantán-González *et al.* 2015). This has led to its use as a biocontrol agent in the agriculture and food industry (Wisniewski *et al.* 1991; Hashem and Abo-Elyousr 2011). Another killer yeast species, *Wickerhamomyces anomalus*, has been suggested as a possible candidate for integrated vector control (Ricci *et al.* 2011; Martin *et al.* 2016); interestingly, yeasts of the *M. guilliermondii* clade possess a similar antimicrobial activity and can be found in insect hosts as well, opening up the possibility of envisioning similar approaches.

Although *C. guilliermondii*, the anamorph of *M. guilliermondii*, is considered safe and classified as a biosafety level 1 organism, it has been described as an occasional opportunistic pathogen in immunocompromised patients (Pfaller *et al.* 2006). It is estimated to be the sixth most frequent nosocomial yeast (Pfaller *et al.* 2006), causing >11% of all episodes of systemic candidiasis (Girmenia *et al.* 2006).

The *Meyerozyma* species complex belongs to the Saccharomycotina CTG clade, a group of yeasts which has been thoroughly studied in the last 40 yr, however its fine phylogenetic structure remains unclear. Taxonomy has been traditionally ruled by phenotypic (*e.g.*, morphologic and metabolic) features, making it a challenging task in yeasts due to the paucity of discriminative morphological characters. More recently, molecular features (*e.g.*, single nucleotide polymorphisms in a single gene or sets of genes) have been widely adopted by taxonomists in general, and this shift clarified a number of phylogenetic relationships in all Kingdoms of life, including Fungi (Kurtzman 1994). Yeast taxonomy, however, remains a complicated matter, with multiple synonyms for each species and an exception to the “one species one name” rule concerning yeasts in different sexual stages (teleomorph/anamorph) (Taylor 2011).

Specifically, *M. guilliermondii*, formerly known as *Pichia guilliermondii*, has recently been placed into its own genus (Kurtzman and Suzuki 2010) and is thought to form a species complex with close relatives *M. caribbica* and *Candida carpophila* (no known teleomorph) (Vaughan-Martini *et al.* 2005). Furthermore, given its emerging pathogen status, it is important to be able to correctly identify yeasts belonging to this species complex, particularly *M. guilliermondii* and *M. caribbica*, the latter of which is less frequent and does not seem to present antibiotic resistance (Pfaller *et al.* 2006). Since a morphological identification within the complex is impossible, several molecular protocols using microsatellites, internal transcribed spacer (ITS) polymorphisms, and ITS restriction fragment length polymorphism (RFLP) fingerprinting, have been developed (Romi *et al.* 2014; Merseguel *et al.* 2015; Wrent *et al.* 2016).

We isolated four yeast strains from the gut of four different Diptera species, namely *Drosophila suzukii*, *Culex quinquefasciatus*, *Anopheles stephensi*, and *Aedes aegypti*. First, we carefully identified them at the species level with RFLP fingerprinting of the ribosomal ITS, one of the most effective molecular protocols for the task (Romi *et al.* 2014). We then performed whole genome sequencing and *de novo* assembled the reads. The resulting genomes were employed for phylogenomic and comparative genomic analyses.

The objective of this study was twofold: first, to examine the genomes of arthropod-associated yeasts, and second, to draw a comprehensive phylogenetic picture of the *M. guilliermondii* species complex exploiting whole genome data. To do so, we integrated our dataset with the reference genomes of the three members of the species complex and one close relative, *Clavispora lusitaniae*, completing it with an already published genome of *Phlebotomus*-associated *M. guilliermondii* (E. Martin I. Varotto Boccazzi, L. De Marco, G. Bongiorno, M. Montagna, unpublished results).

Here we describe these four novel genomes in the context of the *M. guilliermondii* species complex, make them available to the public, and discuss the phylogenetic implications of our results.

## Materials and Methods

### Yeast isolation and characterization

All arthropod samples employed in this study derive from insect colonies maintained at the University of Camerino and at the University of Torino. Diptera were maintained in cages at standard conditions of temperature, humidity, and photoperiod, as previously reported (Ricci *et al.* 2011; Vacchini *et al.* 2017).

The yeast strains derived from the three mosquito species (isolates clone C2, clone 8, and clone 1, respectively, from *C. quinquefasciatus*, *A. stephensi*, and *A. aegypti*), were isolated following a published protocol (Bozic *et al.* 2017). Briefly, homogenized mosquitoes guts were preinoculated in YPD medium (1% yeast extract, 2% peptone, 2% glucose, 2% agar) and suspended in saline solution before plating on selective media 6.5% Sabouraud (Sabouraud powder prepared by the manufacturer, BD Sabouraud Dextrose Agar) with rifampicin 40 µg/ml. Isolate AF2.6.P.231 was obtained from an adult individual of *D. suzukii*. After surface sterilization by washing once with ethanol and twice with deionized water, serial dilutions of the insect homogenate were plated on Potato Dextrose Agar (PDA). Once growth was visible, a colony was purified three times on solid PDA and then conserved at −80°. For identification, DNA was extracted from the isolate using boiling lysis (Marasco *et al.* 2012).

Genomic DNA was extracted from individual yeast samples grown in YPD medium, using JetFlex Genomic DNA Purification Kits (Genomed, Löhne, Germany). Quantity and quality of the recovered DNAs were checked by spectrophotometer and stored at −20°. After incubation for 48 hr at 28°, yeast colonies were subjected to PCR to amplify a polymorphic fragment of the 18S rRNA gene using oligos yeast-F1 and yeast-R1 (Ricci *et al.* 2011), or a region comprising the 5.8 rRNA gene and the two sideward regions, ITS1 and ITS2, using primers ITS1F and ITS4 (Manter and Vivanco 2007). Subsequently, the amplification products were sequenced and BLAST was used to characterize the isolated yeasts at the genus level.

RFLP was performed on all isolates to specifically discriminate between *M. guilliermondii* and *M. caribbica*, as previously described (Romi *et al.* 2014). Briefly PCR amplification using primers ITS1 (5′-TCCGTAGGTGAACCTGCGG-3′) and ITS4 (5′-TC CTCCGCTTATTGATATGC-3′) was carried out to amplify a polymorphic ITS fragment. The PCR product (4 μl) was digested with 5 U of *Taq*I (Promega, Madison, WI) in a 10 μl reaction volume at 65° for 2 hr, as per manufacturer’s instructions. The restriction patterns were analyzed by electrophoresis of the 10 μl reaction volume on 2.0% (w/v) agarose gel.

### Sequencing, assembly, and annotation

Total DNA was sequenced by an external company (Mr. DNA, Shallowater, TX) in 1 run of 2×150 paired-end reads on a HiSeq-2500 platform (Illumina). Reads are available in the European Nucleotide Archives (ENA; https://www.ebi.ac.uk/ena) under the accession numbers ERX2126952–5.

Quality of the raw reads was assessed for each sample using FastQC (Andrews 2010). *De novo* assembly was performed using SPAdes version 3.8.2 (Bankevich *et al.* 2012) employing different k-mer lengths (21, 33, 55, 77, 99, 111), setting the --cov cutoff parameter to auto, and using the --careful option. The assembled contigs of each genome are available at ENA under accession numbers GCA_900231965, GCA_900231995, GCA_900232055, and GCA_900232065 for *C. quinquefasciatus*, *A. stephensi*, *D. suzukii*, and *A. aegypti*, respectively. All genomes analyzed in this study, and in newly sequenced and reference ones, were annotated using the following procedure. First, gene calling was performed using GeneMark-ES Suite version 4.32 (Ter-Hovhannisyan *et al.* 2008) with the parameter –min_contig set to 10,000 and using the –fungus option. Then, Clusters of Orthologous Groups (COGs) (Tatusov *et al.* 2000) were assigned to the obtained translated genes by the COGnitor software (Tatusov *et al.* 2000).

### Genomic analysis

The genomes of members of the *M. guilliermondii* species complex and of *C. lusitaniae* were retrieved from NCBI with the following GeneBank assembly accessions: *M. guilliermondii* (GCA_000149425.1 and GCA_900174495.1), *M. caribbica* (GCA_000755205.1), *C. carpophila* (GCA_001599235.1), and *C. lusitaniae* (GCA_000003835.1).

Orthogroups were inferred with Orthofinder 1.1.4 (Emms and Kelly 2015) from the predicted sets of proteins. Single-copy orthogroups (SCO) were then selected, defined as orthogroups with exactly one protein in all samples, with a custom Python script. Each SCO was aligned using MUSCLE 3.8.31(Edgar 2004), and we consecutively tested each multiple sequence alignment (MSA) for recombination using the software PhiPack (Bruen and Bruen 2005). An SCO group was considered as not having signs of recombination if it passed all three tests run by PhiPack. Then, nonrecombinant SCO MSAs were polished with Gblocks 0.91b (Castresana 2000) and concatenated with a custom Python script. Finally, the concatenated alignment was used as input for RAxML version 8.2.8 (Stamatakis 2014) under the PROTCAT approximation, using the LG substitution matrix and 100 bootstrap replicates. A comparative genomic approach was designed, integrating the functional and phylogenetic data obtained. Phylogenetic clades were analyzed for COG content using in-house Python scripts.

### Data availability

Reads are available in the ENA (https://www.ebi.ac.uk/ena) under the accession numbers ERX2126952–5. The assembled contigs of each genome are available at ENA under accession numbers GCA_900231965, GCA_900231995, GCA_900232055, and GCA_900232065.

## Results and Discussion

### Yeast isolation and characterization

Yeasts were isolated from four insect species and characterized at the species complex level using PCR and Sanger sequencing. 18S fragments sequenced for the three yeasts isolated from mosquitoes were identical and presented 99% sequence identity with an 18S gene belonging to *M. guilliermondii*, GenBank accession number KX258468.1. For the yeast isolated from *D. suzukii*, a fragment of the ITS was sequenced, showing a 100% sequence identity with sequence KU216711.1 of *M. guilliermondii*. These results allowed us to identify the isolates at the genus level; however, in order to discriminate between *M. guilliermondii* and *M. caribbica*, a specific RFLP protocol (Romi *et al.* 2014) was performed, which clearly showed that all novel isolates exhibit the restriction fragment pattern typical of *M. caribbica*. The same RFLP protocol was performed on the yeast derived from *P. perniciosus* (characterized in E. Martin I. Varotto Boccazzi, L. De Marco, G. Bongiorno, M. Montagna, unpublished results), confirming its identification as *M. guilliermondii*.

### Assembly and annotation

Raw reads were high quality for each of the samples analyzed. The four novel draft genomes present a reduced amount of fragmentation (from 43 to 144 contigs longer than 1000 bp) and are sized coherently compared to the reference genomes of the *Meyerozyma* genus (Table 1). It has to be noted that, although the two reference genomes were sequenced with a combination of long and short reads, we only employed a paired-end short reads library, thus obtaining a larger amount of contigs. Nevertheless, GeneMark-ES, a self-training gene calling algorithm, predicted a comparable amount of genes (from 5111 to 5774) for all genomes analyzed (Table 1), including the reference genomes. Additionally, COGnitor assigned a similar number of COGs (from 3288 to 3598) to a similar number of unique genes (from 3029 to 3312) for all sets of predicted proteins (Table 1).

**Table 1:** Assembly and annotation statistics of the four novel genomes (marked with an asterisk) and of the published genomes used for comparative analysis; contigs shorter than 1 kbp were discarded

Species	Isolate	Genome Size, bp	Contigs	Genes	Genes with COG
*M. guilliermondii*	ATCC6260	10,609,954	9	5401	3312
*M. caribbica*	MG20W	10,609,282	9	5390	3305
*C. carpophila*	JCM9396	10,242,926	10	5296	3219
*C. lusitaniae*	ATCC42720	12,114,892	9	5111	3029
*M. guilliermondii*	*P. perniciosus*	10,642,597	31	5487	3362
*M. caribbica*	D. melanogaster*	10,387,257	43	5367	3242
*M. caribbica*	C. quinquefasciatus*	10,553,449	51	5453	3301
*M. caribbica*	A. stephensi*	11,040,470	144	5774	3481
*M. caribbica*	A. aegypti*	10,347,015	48	5359	3237

### Genomic analysis

All analyzed genomes show high similarity for what concerns the inferred orthogroups. Almost the total amount of proteins (98.7%) were assigned to an orthogroup; moreover, out of a total of 5371 orthogroups, 5142 had at least one protein in each genome of the *M. guilliermondii* species complex (all genomes analyzed except the outgroup *C. lusitaniae*), whereas 4050 had all species represented, indicating the presence of a strong core set of genes.

**Figure 1:**
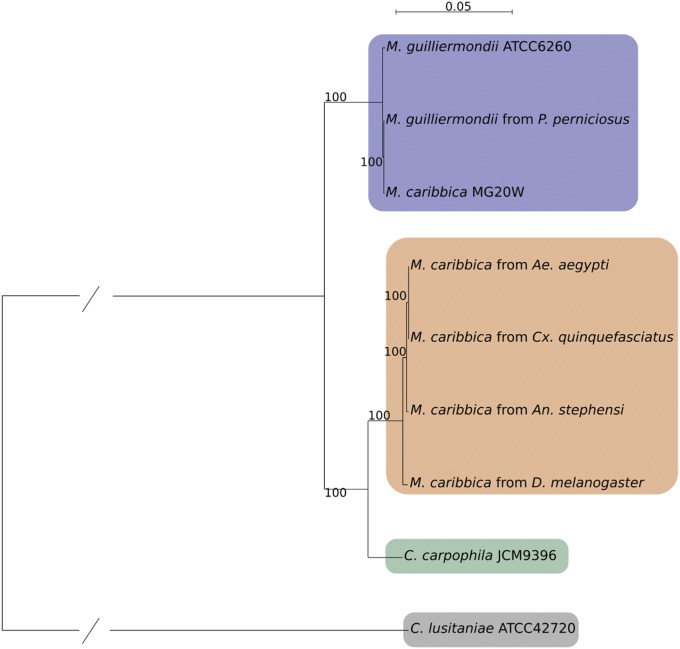
Maximum likelihood tree. Resulting clades are highlighted: *M. guilliermondii* (blue), *M. caribbica* (orange), *C. carpophila* (green), and *C. lusitaniae* (gray).

We retrieved 3408 SCOs, which were then aligned with MUSCLE and tested for recombination with PhiPack. We retained 2147 nonrecombining SCOs and processed their MSAs with Gblocks. The polished MSAs were concatenated obtaining an 870,212 bp long alignment. We used this final MSA as input for RAxML, obtaining a phylogenetic tree with 100% bootstrap support for all branches (Figure 1). Four clear clades can be seen in the tree: (1) *C. lusitaniae* as the outermost single species clade, (2) the reference genomes of *M. guilliermondii* and *M. caribbica* clustering together with the *M. guilliermondii* genome isolated from *P. perniciosus* in a clade with reduced branch lengths, (3) *C. carpophila* as a single-species clade, and (4) all novel *M. caribbica* genomes clustering together in a clade with reduced branch lengths, closer to the *C. carpophila* clade than to the reference *M. guilliermondii*/*M. caribbica* clade.

This is the first phylogenetic study attempting to describe the *M. guilliermondii* species complex employing whole genome sequencing data. The genome of *M. guilliermondii* has already been included in a phylogenomic analysis in a study which, among other things, clarifies its position inside the CTG clade (Butler *et al.* 2009). In this work we expand the genomic dataset of the *M. guilliermondii* species complex, including genomes of *M. caribbica* and *C. carpophila*. Literature on these two species is scarce compared to *M. guilliermondii* and past phylogenetic inferences were based on single or few genes and did not include all members of the species complex, leaving doubts about fine characterization (Kurtzman and Suzuki 2010; Kurtzman and Robnett 2013). Considering these facts, it would have been presumptuous to make assumptions on the phylogenetic structure of the complex.

Our phylogenetic tree has one glaring issue: the position of the reference genome of *M. caribbica* (Figure 1). At first sight, its closeness to the genome of *M. guilliermondii* would not be suspicious since they share the genus name and they are notoriously difficult to differentiate, even with traditional molecular markers. The aforementioned difficulty to discern the two species, in our opinion, is the key to solve this phylogenetic enigma, considering two facts: (1) the reference genome of *M. caribbica* is the sister group to the genome of *M. guilliermondii* isolated from *P. perniciosus*, and (2) our genomes of *M. caribbica* isolated from arthropods form a distinct clade, closer to *C. carpophila*. If we accept the identification as *M. caribbica* for the isolate previously sequenced (Kim *et al.* 2015), it would seem that two distinct *M. caribbica* exist: one is phylogenetically indistinguishable from *M. guilliermondii* whereas the other forms a distinct clade, sister group to *C. carpophila*. We think that the most likely and parsimonious explanation is that the reference genome of *M. caribbica* has been misidentified and actually belongs to *M. guilliermondii*.

**Figure 2:**
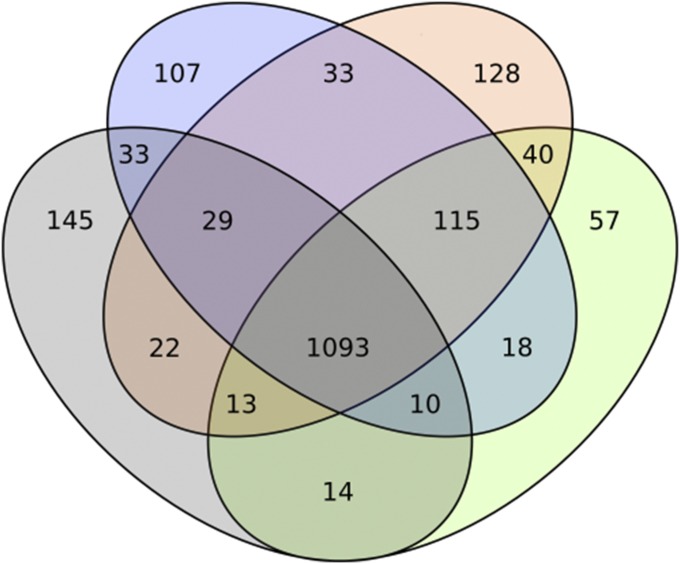
Venn diagram representing COG content in the clades resulting from the phylogenomic analysis: *M. guilliermondii* (blue), *M. caribbica* (orange), *C. carpophila* (green), and *C. lusitaniae* (gray).

As a follow-up to the phylogeny, the COG content of the four described clades was compared (Figure 2). In total, 1093 COGs are shared between the four clades, constituting the vast majority of COGs assigned to each clade. This result confirms the evolutionary similarity of all analyzed genomes and a probable corresponding functional closeness. The outgroup, *C. lusitaniae*, holds the most unique COGs at 145 followed by the *M. caribbica* clade (128); the *M. guilliermondii* clade has a comparable number of unique COGs (107) whereas *C. carpophila* presents with the least (57). We think that these results confirm the phylogenetic distance between the *M. caribbica* clade and *M. guilliermondii* clade observed in the phylogenetic tree.

### Conclusions

Yeasts of the *M. guilliermondii* species complex are relevant for multiple reasons. They are widely used in industry due to their useful properties, have recently emerged as nosocomial pathogens and, due to their proprieties, they can be envisioned as potential tools for the control of arthropod-borne diseases. Our study provides a useful genomic resource and a detailed phylogenomic analysis of this species complex, providing novel insights into the evolutionary history of these yeasts. Additionally, we provide four novel genomes belonging to *M. caribbica*, arguably the first of this species.
